# Supplementary material for: Metal-Organic Framework Assembled on Oriented Nanofiber Arrays for Field-Effect Transistor and Gas Sensor-Based Applications
Source: Molecules. 2022 Mar 25;27(7):2131. doi: 10.3390/molecules27072131 (PMC9000462; doi:10.3390/molecules27072131)
Supplement: Supplementary file 1 [file molecules-27-02131-s001.zip › molecules-1616289-supplementary.pdf]

Supporting Information

for

In-Situ Grown Metal-Organic Framework on Oriented Nanofiber Arrays for Field-Effect Transistor and Gas Sensor-Based Applications

Jinlong Mu, Xing Zhong, Wei Dai, Xin Pei, Jian Sun, Junyuan Zhang, Wenjun Luo * and Wei Zhou *

Engineering Research Center of Nano-Geomaterials of Ministry of Education, Faculty of Materials Science and Chemistry, China University of Geosciences,388 Lumo Road, Wuhan 430074, P. R. China
mujl1122@foxmail.com (JL.M); 2405704360@qq.com (X.Z); 1552840502@qq.com (W.D); 1438387289@qq.com (X.P); sunjian@cug.edu.cn(J.S); 25811481112@qq.com (JY.Z);luowj@cug.edu.cn (WJ.L);
weizhou@cug.edu.cn (W.Z)
* Correspondence: luowj@cug.edu.cn (WJ. L); weizhou@cug.edu.cn (W. Z); Tel.: +86 159 2649 7061; fax: +86 027 6788 3740

Section S1. Materials and Methods

All chemicals were purchased from commercial sources and used without further purification unless otherwise specified.

Fourier transforms infrared (FT–IR) spectra measurements were performed on an instrument (EQUINOX55, Bruker, Germany) using the standard KBr disk method. Surface morphology and energy dispersive X-ray spectroscopy (SEM/EDX) analyses were examined on a field emission scanning electron microscope (FE-SEM, FEISirion200, Philip, Netherlands), operated at 20 kV. X-ray diffraction (XRD) patterns were carried out on a Netherlands PANALYTICAL powder diffraction system with Cu target operating at 45 kV and 50 mA. Transmission Electron Microscope (TEM, JEOL JEM-F200, Japan) operated at an accelerating voltage of 200 kV, X-ray photoelectron spectrometer (XPS, MULTILAB2000, U.S.A.) was used in the surface analysis of samples with Al Kα radiation operating at 1486.6 eV. Nuclear Magnetic Resonance (NMR) spectra were recorded on Bruker Avance NEO 600 (Germany). Solvent signals were used as internal standards for chemical shifts (1H: = 7.26 ppm for $CDCl_3$), (^1H: 600 MHz, ^{13}C: 150 MHz). V-I was measured by KEITHLEY2400 Source Meter Hall Effect Measurement System. A flat capacitor (Al/ PMMA: PDMS-PCDA/Cu/ Al) was prepared using the composite dielectric film (20 % PMMA: 80 % PDMS-PCDA/Cu). We selected the capacitance value and permittivity of the capacitor at a frequency of 30kHz. Optical images were acquired with AxioScope A1 (Carl Zeiss). Optical photos were captured by a mobile phone. Crystal structures were drawn by the software of VESTA.

Field Effect Transistors measurements

Electronic properties were measured by a KEITHLEY4200-SCS semiconductor characterization system. The field-effect transistor was fabricated on an (n++) Si-wafer with a top-contact configuration. Distance between two Au electrodes is L = 0.075 mm; Side-length of the electrode is W= 2.525 mm. The charge mobility calculated by the field-effect was based on the following formula (Cgate is the capacitance of PMMA: PDMS-PCDA/Cu layer 11.2 nF/cm^2 in Figure S5):

$$\mu = \frac{dDrainI}{dGateV} \cdot \frac{L}{W} \cdot \frac{1}{Cgate \cdot DrainV}$$

Gas sensor characterizations

$Cu_3(HITP)_2$ NFAs -based chemiresistive sensors were fabricated using silver paste as the electrode, and 50 μm diameter nickel wire attached to the silver paste as the conducting wires. The sensors were measured in a gas sensing system (Wison Electronic Technology Co., LTD, WS-30B). The system adopts the current and voltage test method, and the primary test principle is shown in Figure S7. The sensor devices were placed in a sealed quartz chamber at room temperature, and dry air was used as the carrier gas for the target gas. The voltage changes of the device under different concentrations of target gas were monitored to conduct gas sensing experiments.

The solution of the target gas was introduced into the heating table of the quartz tube through the quantitative needle. All of the sensing measurements were performed at ambient conditions.

Section S2. Chemical Synthesis

Synthesis of PDMS-PCDA Ligand

PDMS-PCDA was synthesized according to a known report; all chemicals were purchased from commercial sources and used without further purification unless otherwise specified. (Et_3N was added to a solution of H_2N-PDMS-NH_2 (Mn = 6000) in anhydrous CH_2Cl_2 at 0 °C under argon atmosphere. After stirring for 2 h, a solution of 2,6-pyridinedicarbonyl dichloride in CH_2Cl_2 was added dropwise. The resulting mixture was stirred for 2 h while the temperature was kept at 0 ℃ with ice water. The solution was then allowed to warm to room temperature and stirred for two days. After the reaction, MeOH was poured into it to quench the reaction. The final product was subjected to vacuum evaporation to remove

the solvent and trace of Et_3N. ^1H NMR (400 MHz, $CDCl_3$): δ 8.36 (d, J = 8.0 Hz, 2H), 8.02 (t, J = 8.0 Hz, 1H), 7.74 (s, 2H). ^{13}C NMR (400 MHz, $CDCl_3$): δ 163.57, 149.17, 139.1, 125.17.

Preparation of PDMS-PCDA/Cu NFAs

0.8 g PDMS-PCDA was added into 2 ml methylene chloride for thorough stirring. A certain amount of copper sulfate pentahydrate was added into methanol for thorough stirring by calculation. The two solutions were stirred magnetically in a sealed glass bottle for two days and then in the air for 24 h. The mixture was put into a vacuum drying oven at 80 ℃. After vacuum drying for 24 h, the uncoordinated copper ions were removed by the repeated dissolution of dichloromethane and methanol. 0.2 g PMMA was dissolved in 4ml tetrahydrofuran（THF） for thorough stirring for 8 h, and PDMS-PCDA /Cu was added. The solution was transferred to a 20mL syringe after 5h of stirring and 4 ml of dimethylformamide (DMF) for 2 h of stirring. The parameters of electrostatic spinning were adjusted (voltage 18 kV, acceptance distance 15 cm, propulsion speed 0.2 mL / h). The (n++) Si substrate was fixed on the drum, and the drum speed was set to 300 r/min, 400 r/min, and 500 r/min, respectively. After receiving the PDMS-PCDA/Cu nanofiber arrays, the substrate was placed in a vacuum drying line for 10 h for reserve.

Synthesis of $Cu_3(HITP)_2$ power and $Cu_3(HITP)_2$ NFAs

$Cu_3(HITP)_2$ power was synthesized according to a known report.

$Cu_3(HITP)_2$ NFAs: PDMS-PCDA/Cu NFAs on the (n++) Si substrate was first immersed in the solution of aqueous ammonia for 1 h. Under air, the substrate was transferred to a solution of HATP.6HCl in distilled water for 1 h, washed by distilled water, and dried. Then it was added to a solution of $CuSO_4.5H_2O$ in distilled water and concentrated aqueous ammonia at room temperature for 1 h, washed by distilled water, and dried. Follow these steps 20 cycles to obtain $Cu_3(HITP)_2$ NFAs.

Section S3. Characterization

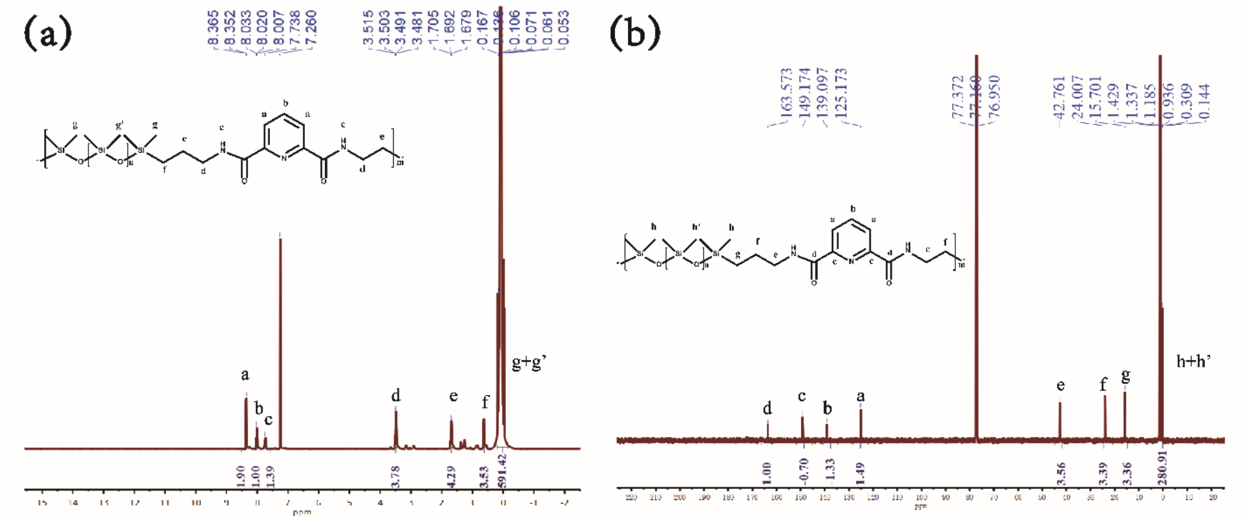

Figure S1. ^{1}H NMR (a) ^{13}C NMR (b)spectra of PDMS-PCDA

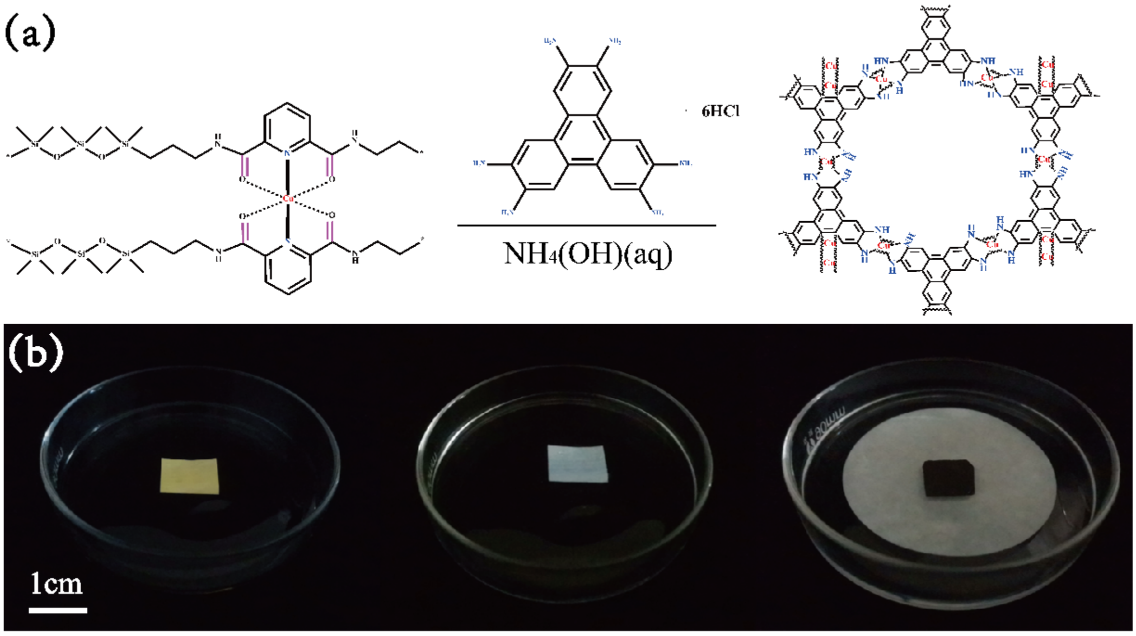

Figure S2. (a) Technology route of $Cu_3(HITP)_2$fiber membrane preparation (b) Process of change from PDMS-PCDA/Cu fiber membrane to $Cu_3(HITP)_2$ fiber membrane

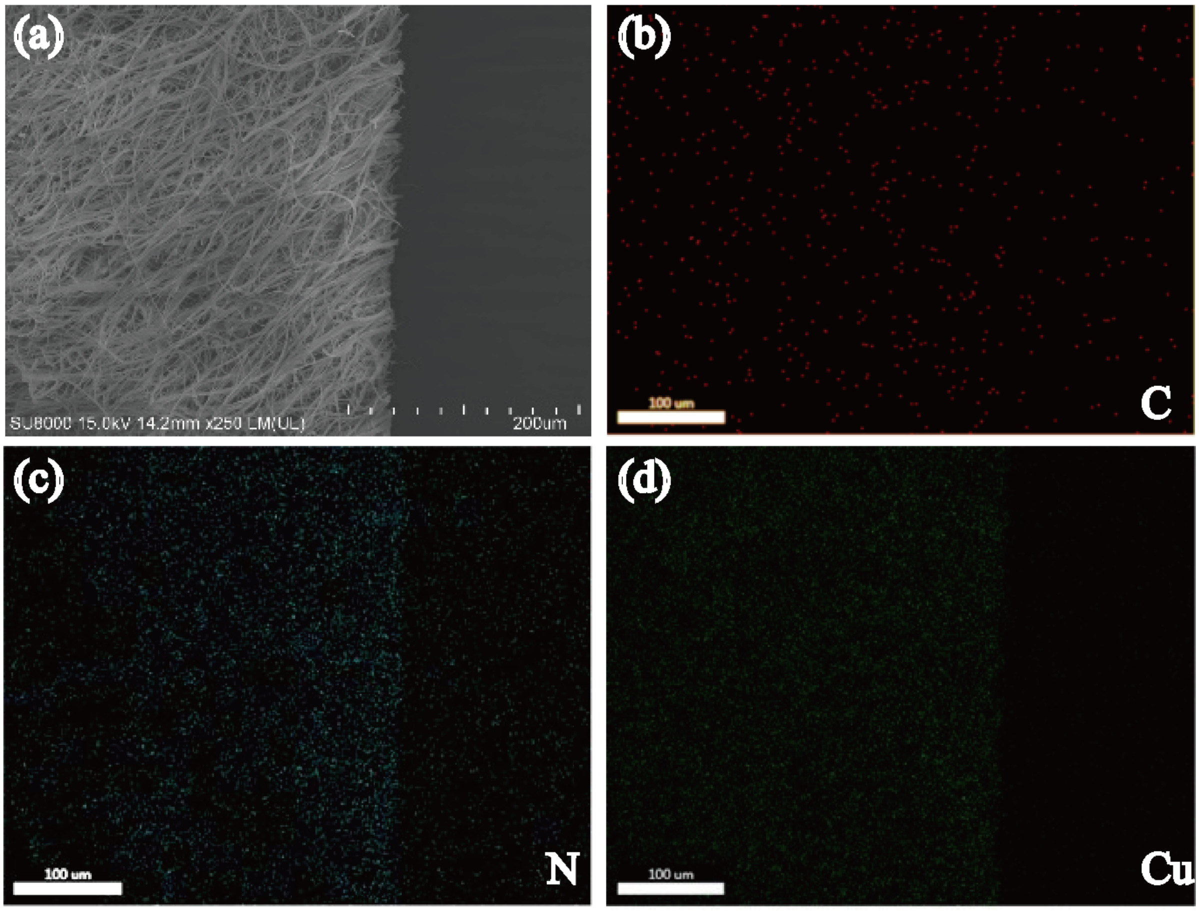

Figure S3. (a)SEM image, and corresponding EDS elemental mapping of the $Cu_3(HITP)_2$ fiber membrane: (b) C, (c) N, (d) Cu elements.

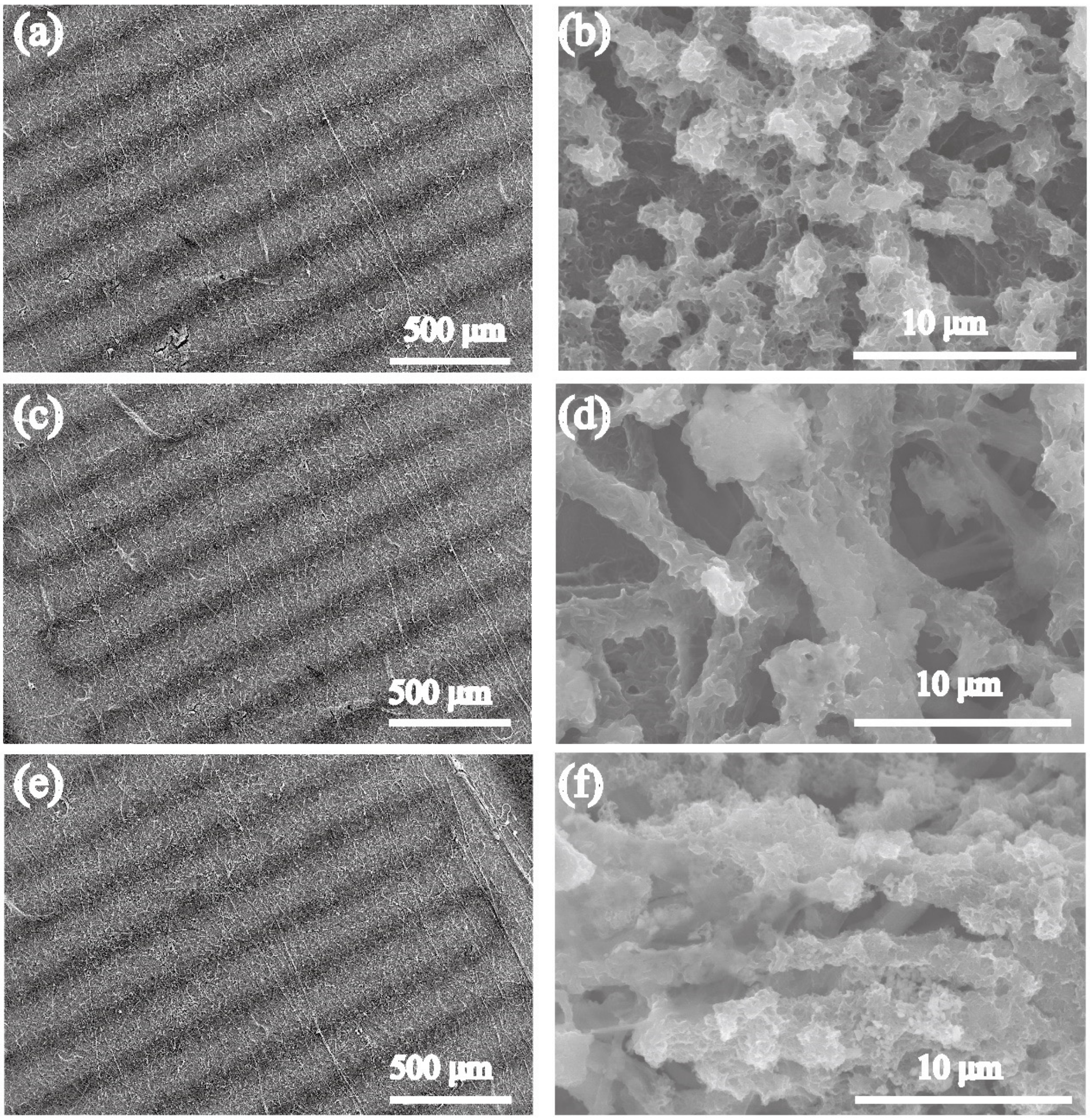

Figure S4. SEM images of $Cu_3(HITP)_2$ NFAs-based FET: The Au electrode evaporated on the $Cu_3(HITP)_2$ NFAs of 300 r/min (a), 400 r/min (c), 500 r/min (e), and the corresponding interelectrode fiber arrays (b, d, f).

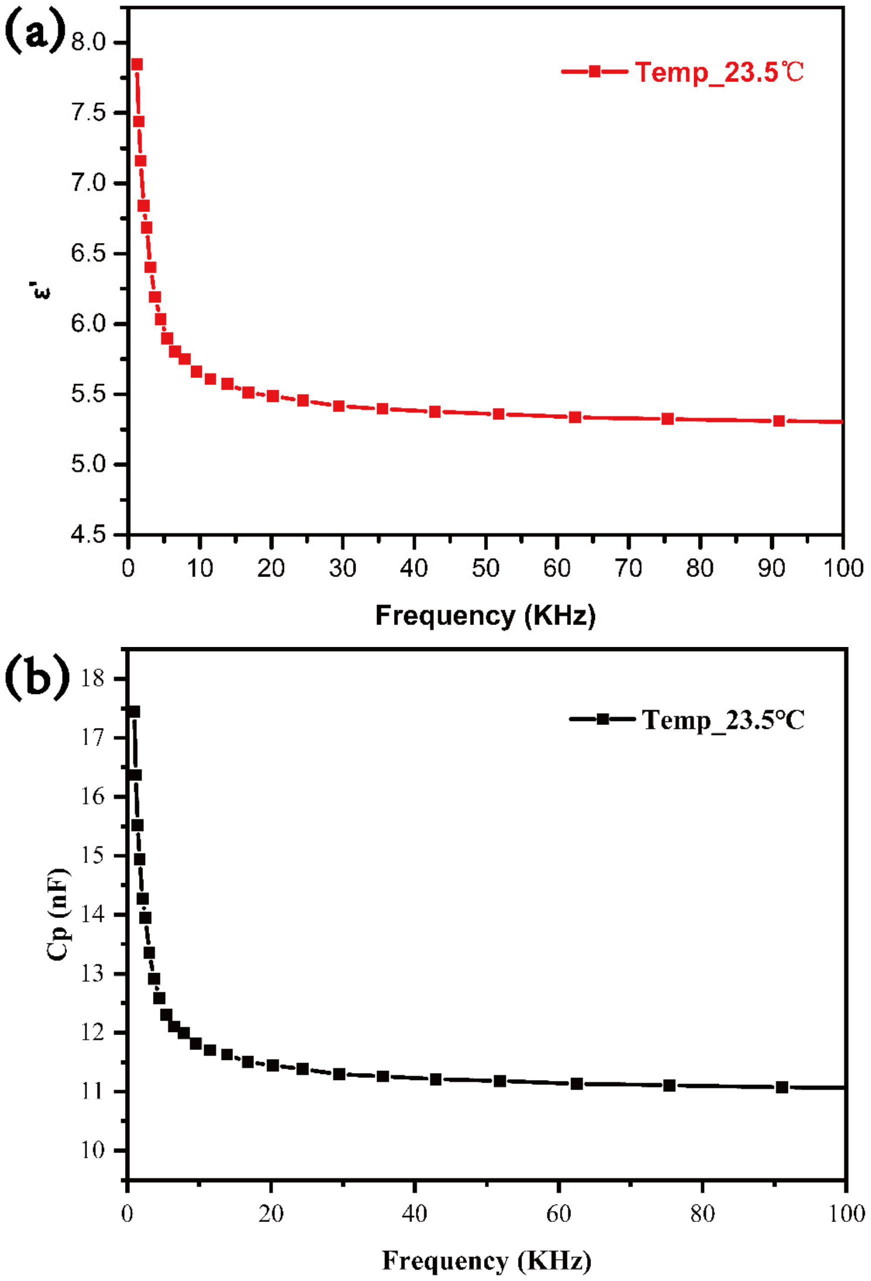

Figure S5. Capacitance value and permittivity of the composite dielectric film (20 % PMMA: 80 % PDMS-PCDA/Cu).

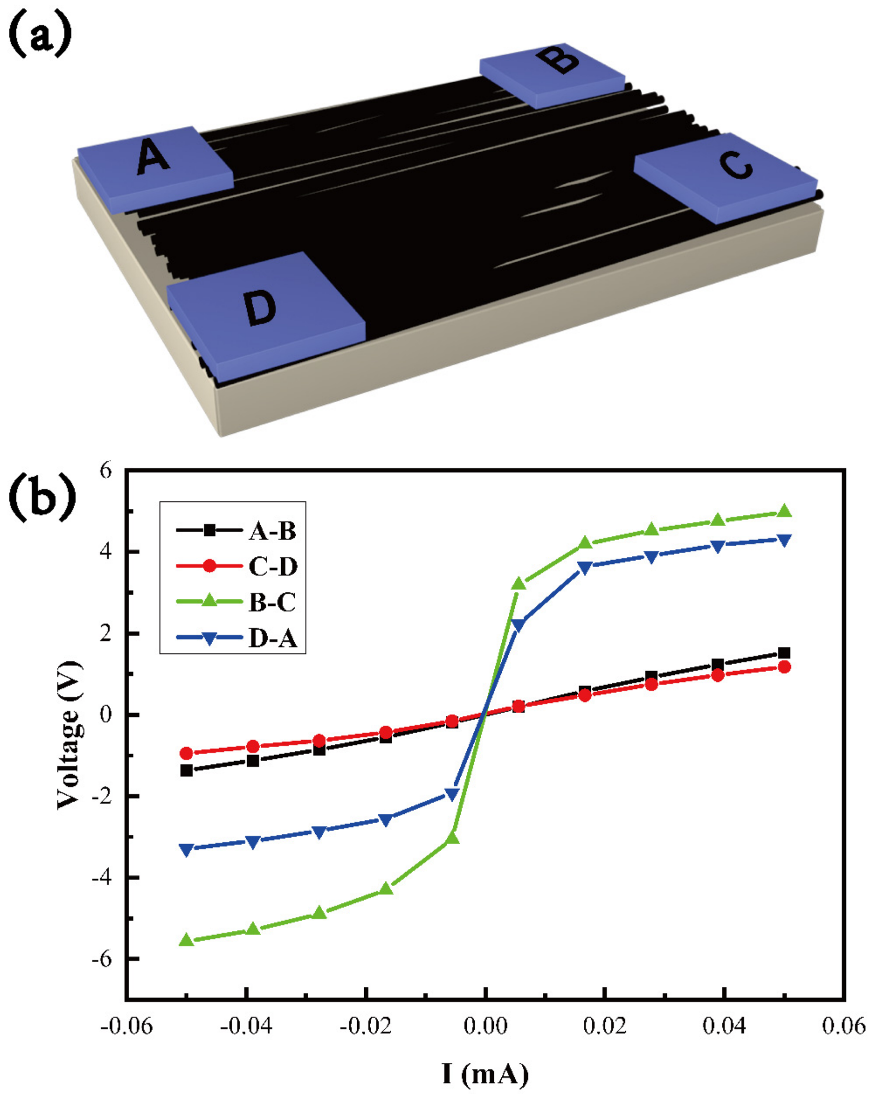

Figure S6. (a) Schematic illustration of $Cu_3(HITP)_2$ NFAs (500 r/min) and (b)V-I curves (I_S=0.05 mA).

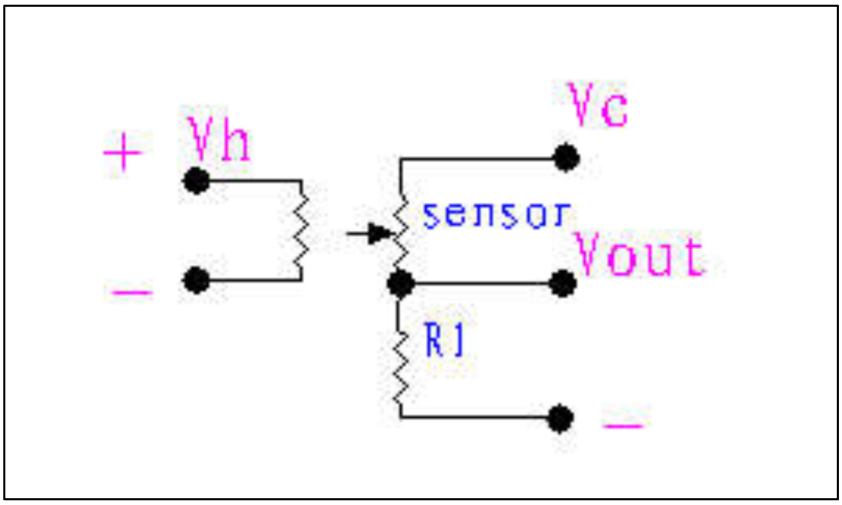

Figure S7. Working principle of the gas-sensitive element test system

The system adopts the current and voltage test method. V_h: the working heating power supply, Vc: Loop power supply; The characteristics of the gas-sensitive element are analyzed by testing the voltage V_{out} on the load resistance R_l in series with the gas-sensitive element.
